# Experimental Study on the Permeability of SAP Modified Concrete

**DOI:** 10.3390/ma13153368

**Published:** 2020-07-29

**Authors:** Yaohua Guo, Puyang Zhang, Hongyan Ding, Conghuan Le

**Affiliations:** 1 State Key Laboratory of Hydraulic Engineering Simulation and Safety, Tianjin University, Tianjin 300072, China; guoyaohua@tju.edu.cn (Y.G.); dhy_td@163.com (H.D.); leconghuan@163.com (C.L.); 2 Key Laboratory of Coast Civil Structure Safety, Ministry of Education, Tianjin University, Tianjin 300072, China; 3 School of Civil Engineering, Tianjin University, Tianjin 300072, China

**Keywords:** superabsorbent polymer (SAP), permeability, concrete, internal pore characteristics, threshold effect

## Abstract

To study the permeability of superabsorbent polymer (SAP) modified concrete and the effect of internal pore characteristics on the permeability of concrete specimens, the results of the water penetration under pressure test, the mercury intrusion porosimetry (MIP) test, and scanning electron microscopy (SEM) of SAP concrete were obtained and analyzed. The research shows that the addition of an appropriate amount of SAP can effectively improve the anti-permeability performance of concrete. After adding 0.2~0.6% SAP of cement quality to concrete, the penetration height value was reduced by 35~45%, the porosity was increased by 21–95%, and the tortuosity is increased by 14–15%, and all indicators show regular changes with the increase in SAP usage. Adding SAP to concrete changes the internal connection state of concrete, thereby further improving its impermeability by reducing the capillary pressure and changing the shape of the pores. The liquid permeation resistance is increased by the “threshold effect” inside concrete; this “threshold effect” is caused by the addition of SAP.

## 1. Introduction

With the improvement of the aesthetic and functional requirements of building structures, high-strength concrete is increasingly used in modern civil engineering structures. However, high-strength concrete also faces the problem of poor permeability due to its extremely high density, which in turn is difficult for conventional external curing methods to effectively maintain. Using a certain method or additive to achieve and improve the internal curing efficiency of concrete is a feasible solution. Shideler and A. Bentur studied the mechanical properties and volume stability of concrete mixed with pre-wet light aggregate and found that this method helps to reduce the self-shrinkage and cracking of concrete [1,2]. Jensen and Hansen proposed the method of using superabsorbent polymer (SAP) as an internal curing material [3]. The research results of Pang et al. showed that adding SAP to concrete can effectively reduce the self-shrinkage of concrete, and the reduction rate can reach more than 50%, with little effect on the strength of the concrete [4].

Superabsorbent polymers (SAPs) are multi-functional polymer materials. SAP has been widely used in industry, agriculture, forestry, medical and health supplies, and other fields. In the field of civil engineering, the performance of concrete can be improved by a variety of methods that involve the use of SAP as a new type of concrete admixture. SAP can be used as an internal curing material for low water cement ratio concrete. After absorbing water, the SAP particles become distributed uniformly in concrete, thus effectively providing the hydration water in the course of concrete hydration as well as effectively reducing the autogenous shrinkage of concrete and preventing concrete cracking [5,6,7]. SAP can also be used as an air-entraining agent, thereby improving the freeze-thaw resistance of concrete [8,9,10,11]. SAP particles with “water storage” characteristics are helpful for improving the self-healing ability of concrete [12,13]. The addition of the proper amount of SAP to this cement-based material can effectively improve the characteristics of the concrete, thereby widening the application field of concrete.

Concrete is a porous, heterogeneous composite material at various scales. The external media, such as water and gas, are transported in various forms within the material, mainly through its own connected pores and fractures. The permeability depends mainly on the connectivity of the internal pores and the tortuous nature of the infiltration path.

After different stages of the hydration reaction, the internal pore radius of the pores inside concrete is uneven. The difference in capillary pore diameter makes the transport mechanism of gas and liquid through concrete different. In general, when there is a pressure difference, the transport of gas and liquid can be analyzed according to the laws of gas or hydrodynamics (viscous flow, molecular flow, diffusion flow). When there is a difference in concentration, the liquid and the substance dissolved in the liquid will move under the action of diffusion. When there is a potential difference, a phenomenon related to electroosmosis and electrodeposition occurs. When there is a temperature difference, the liquid moving through the concrete is subjected to the thermal mass transfer law.

Water is the element that most commonly and easily comes into contact with the concrete medium. Water molecules can easily enter the concrete through its pores to reduce the pH value of concrete pore fluid. In addition, the water carries harmful ions, such as CL^−^ and SO_4_^2−^ resulting in corrosion of concrete reinforcement, alkali aggregate reaction, and, eventually, the destruction of concrete; thus, to some extent, the permeability of ions determines the rate of deterioration of concrete. Therefore, the study of the permeability of concrete is of great significance. The Rose study found that the main factors that affect the speed and scope of water penetration in concrete materials are the pore structure and the fracture morphology of concrete materials [14]. To study the permeability of superabsorbent polymer (SAP) modified concrete and the effect of the internal pore characteristics on the permeability of concrete specimens, the results of the water penetration under pressure test, the mercury intrusion test, and scanning electron microscopy (SEM) of SAP concrete were obtained and analyzed.

## 2. Internal Curing Mechanism and Mix Proportion

### 2.1. Internal Curing Mechanism

**Figure 1:**
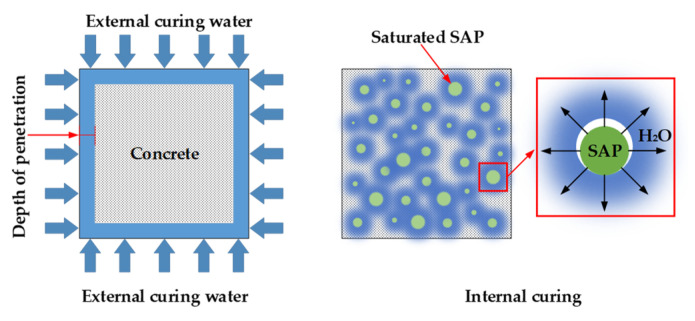
Different mechanisms between external and internal curing methods.

At present, there are two types of concrete curing methods: external curing and internal curing. Traditional curing methods include wet curing (spraying water, covering with wet straw bags, etc.), chemical curing, and the cementing of plastic film on the surface of cement materials. However, these external curing methods are labor-intensive and time-consuming, and because water in external curing has difficulty entering the interior of the concrete, the water required for the subsequent hydration of the cement cannot be continuously replenished, so the final curing effect is poor [15,16]. Internal curing is a special curing method which has the benefit of improving the performance of concrete and thus improving its durability. The different mechanisms of the external and internal curing methods are shown in Figure 1.

According to 2010 ACI Concrete Terminology (American Concrete Institute), internal curing refers to the following: in a fresh cementitious material, the internal water source is provided by a pre-wetted lightweight aggregate (LWA) or a superabsorbent polymer (SAP); the released moisture can be supplied to the hydrating cement [17]. The use of the internal curing material results in the release of water from the powder of concrete via two methods: (1) the pressure difference and (2) the humidity difference between the internal capillary of the cement hardened paste and the internal curing material. Due to the self-drying phenomenon, there is a pressure difference between the concrete pores and the inner pores of the inner curing material, and the water flows from the inner curing material to the cement hardened paste under the pressure difference. According to the Fick law, the moisture of the inner curing material diffuses to the cement hardened paste via the difference of humidity, and the flow rate of diffusion is proportional to the relative humidity gradient. The greater the relative humidity gradient between the two, the greater the diffusion flow; in this manner, the effects of internal curing materials will be more significant.

### 2.2. Properties of SAP Curing Material

According to the different mechanisms of water absorption and storage, the curing materials are divided into two categories. One is a porous structure of light aggregate, mainly including clay or shale quality materials, which can use their own pores to absorb and preserve water; however, its water absorption capacity is low. The other is a superabsorbent polymer, which is a typical functional polymer material with high water absorption; it uses the chemical bond between polymer molecules and water molecules to absorb and retain moisture. The two types of internal curing materials have different structural characteristics, and their mechanisms of interaction with water are quite different; thus, the curing effect of concrete is also different. In this paper, the effect of the latter (SAP) on the impermeability of concrete is studied.

A superabsorbent polymer is a type of polymer material with a strong water absorption capacity and water holding capacity [18,19]. In view of the chemical structure, a strong hydrophilic group, such as a carboxyl group (–COOH) and a hydroxyl group (–OH), is contained within the main chain and the graft side chain of the superabsorbent resin; this hydrophilic group is the main reason for the super absorbent characteristic of the resin.

**Figure 2:**
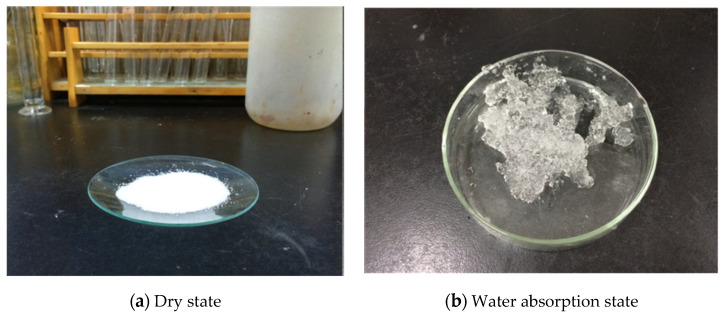
Superabsorbent polymers.

SAP can react with water via the carboxyl group and hydroxyl group and absorb liquid water, which is several thousand times its own weight. SAP exhibits a gelatinous form after absorbing water, as shown in Figure 2. SAP internal water is fixed in the polymer chain by hydrogen bonds, and water is contained in the polymer network structure. The evaporation of water requires a relatively large amount of energy, thereby slowing the water loss caused by evaporation [20,21,22]. Since SAP has a low degree of cross-linked three-dimensional network structure, free water can be immobilized within the polymer network by swelling; this makes it different from the general absorbent material. In addition to a strong water absorbing capacity, even in external physical extrusion, the absorption of water inhibits natural overflow, resulting in a strong water retention capacity.

**Figure 3:**
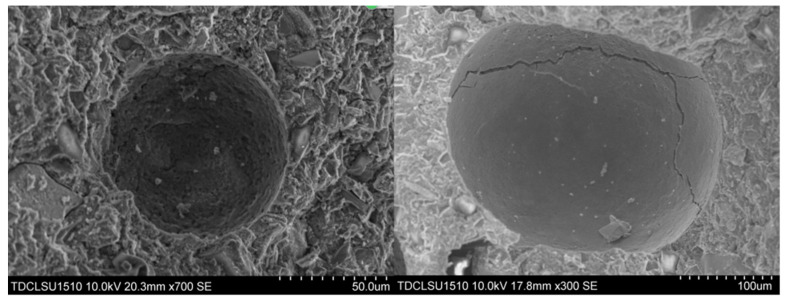
Superabsorbent polymer (SAP) particles and formed pores (SEM).

After SAP water swelling, it will release the corresponding water with an increase in pH value or ionic concentration. In the process of cement hydration, the hydration products of cement produce a large number of cations dissolved into the water, thereby increasing the pH value of the system rapidly; then, the water-containing SAP is continuously released to provide water for the cement to further generate water splash reactions. SAP in the fresh concrete will undergo swelling and form spherical pores in the concrete after drying, as shown in Figure 3; the edge of the spherical pore causes less internal stress concentration.

### 2.3. Mix Proportion of SAP Concrete

The SAP used in this test was acrylic superabsorbent resin (Shanghai Fenghan Chemical Co., Ltd., Shanghai, China), which is based on acrylamide (CH_2_=CH–CONH_2_) as the main monomer, acrylic acid (CH_2_=CH–COOH), methacrylic acid (CH_2_=C(CH_3_)COOH), methyl acrylate (CH_2_=CH-COOCH_3_), ethyl acrylate (CH_2_=CH–COOCH_2_ CH_3_) and methacrylate (CH_2_=C(CH_3_)COOR) as comonomers, and then binary polymerization. The particle sizes of the SAP were 50 mesh and 100 mesh, the performance index of which is shown in Table 1. SAP was added in concentrations of 0.2%, 0.4%, and 0.6% of cement quality, the water-cement ratio was 0.3, and 7 sets of contrast tests were designed. The concrete mix ratio is shown in Table 2.

The additional water diversion in the test was determined by the Powers model [23]; the model quantitatively describes the change in the volume fraction of each phase in the hydration process. The volume of the hydration product is smaller than the sum of the volume of cement and water; this phenomenon of a decrease in the total volume of the cement hydration product is called chemical shrinkage. The volume reduction in the cement hydration reaction is approximately 6.4 mL/100 g. When the capillary water is exhausted, the hydration reaction gradually slows down until, finally, the hydration stops. The complete hydration condition for 1 g of cement requires approximately 0.23 g of bound water and 0.19 g of gel water. Thus, complete and thorough hydration can only occur when W/C ≥ 0.42. When W/C < 0.42, the process of cement hydration must absorb water from the surroundings [3,18]. This will inevitably lead to chemical shrinkage on the micro scale (resulting in a reduction in volume) and will also inevitably lead to concrete drying on the macro scale. Free water will only ensure maximum hydration of the cement and does not guarantee that all the cement can be hydrated.

According to the Power model, the formula for calculating the amount of internal curing water is as follows:

(1) $$W_{e}/C=\{\begin{array}{c} 0.18W/C\;for\;W/C\leq 0.36\\ 0.42-W/C\;for\;0.36\leq W/C\leq 0.42 \end{array}$$

where *W*/*C* represents the water–cement ratio, and *W*_e_/*C* represents the ratio of the additional water diversion to cement quality.

The water–cement ratio of concrete in this experiment is 0.3; thus, the calculation of additional water diversion is as follows:

*W*_e_/*C* = 0.18 × 0.3 = 0.054, *W*_e_ = 0.054 × 550 = 29.7

The strength grade of the cement used in this experiment is 42.5 Portland cement, which represents the minimum value of the standard value of the compressive strength of the cement test piece, 28 d, which is 42.5 MPa. The concrete mix proportion in the experiment is shown in Table 2.

SAP is extremely water-absorbent; it tends to clump after absorbing water and is difficult to stir evenly. Therefore, this test uses a dry mixing method for mixing. First, SAP was mixed with cement, sand, and pebble. After mixing evenly, water was added 3 times by spraying and the mixture was stirred evenly.

## 3. Penetration and MIP Experiment

### 3.1. Experimental Program

**Figure 4:**
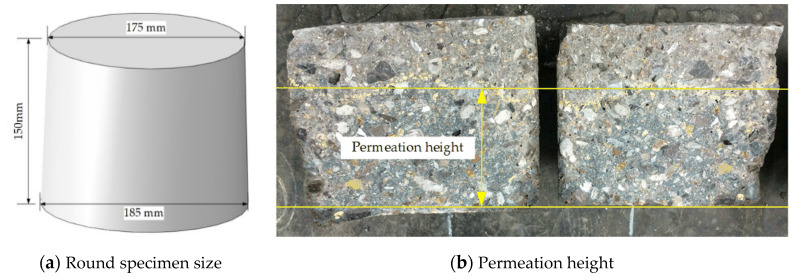
Permeation specimens and results.

According to the different particle sizes and the amounts of SAP, the specimens were divided into 7 groups; the test groups are given in Table 3. Based on the water penetration under pressure test using the penetration depth method, the relative permeability coefficient was calculated by measuring the seepage height of SAP concrete under constant water pressure, and the impermeability of the different types of concrete is compared; the testing process is shown in Figure 4. The water penetration under pressure test specimens were round specimens with a diameter of 175 mm, a bottom diameter of 185 mm, and a height of 150 mm, as shown in Figure 4a. The experimental equipment was an HS-40 concrete infiltration apparatus; the average values of a set of six test pieces were taken as the test results. The pore structure parameters of concrete were obtained via an MIP (mercury intrusion porosimetry) experiment; the test equipment used in the MIP experiment was an Auto Pore IV 9500 manufactured by the United States Micromeritics Company (Norcross, GA, USA). The instrument can be used for low-pressure and high-pressure analysis: the pressure of low-pressure analysis is 3.45–310 kPa, while that of the high-pressure analysis is 227.527 kPa, and the measurement range is 360–3.6 µm.

The osmotic coefficient is calculated according to Formula (2):

(2) $$K=\frac{\phi D_{m}^{2}}{2TH}$$

where *K* represents the osmotic coefficient (units: cm/h), *D*_m_ represents the average water penetration height (unit: cm), *H* represents the water column height (unit: cm), *T* represents the testing time (unit: h), and *ϕ* represents the porosity of concrete, which can be obtained using an MIP experiment.

### 3.2. Water Penetration under Pressure Test Results

**Figure 5:**
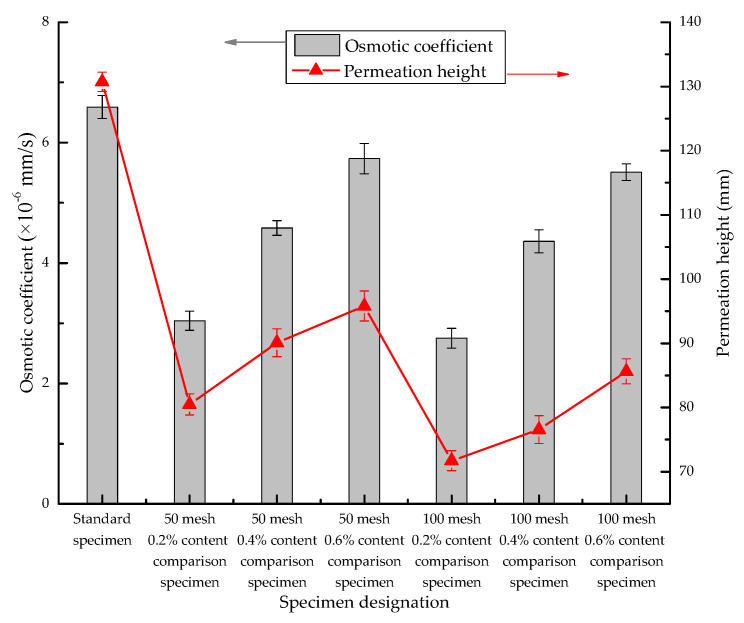
Relationship between osmotic coefficient Kr and permeation height.

Permeation experiments were conducted on each group of samples to obtain the penetration height and calculate the corresponding osmotic coefficient; the calculated results are shown in Figure 5. Figure 5 reveals that the anti-penetration efficiency is better for specimens P-50 and P-100 compared with specimen P-0. The permeation height and permeation coefficient also increased with the increase in SAP content but were still lower than those of the standard sample without SAP. The anti-permeability of the P-50 and P-100 series increases with the increase in SAP content, with the best anti-permeability performance observed in the P-50-0.2 and P-100-0.2 sample groups. The results show that the anti-permeability of concrete can be obtained with a low dosage of SAP. The anti-permeability decreases with the increase in dosage, but it is still higher than that of ordinary concrete.

**Figure 6:**
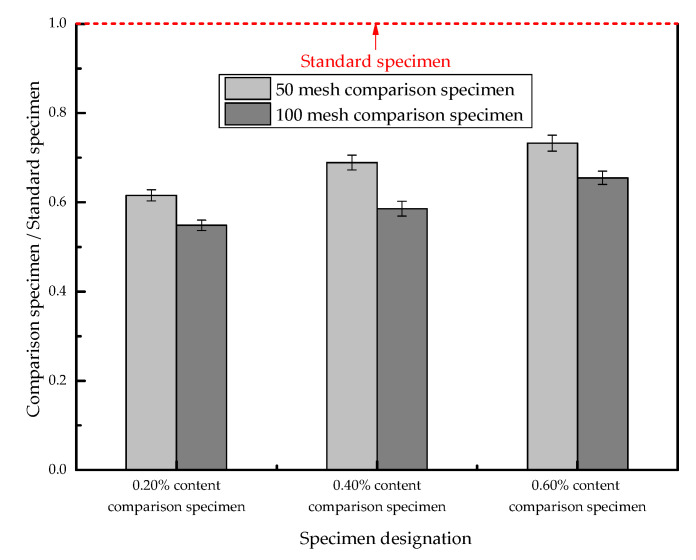
The normalized contrast map of penetration height.

The permeation heights of the P-50 and P-100 samples are normalized by the penetration height of the P-0 samples; the normalized infiltration heights of the samples are shown in Figure 6. Figure 6 reveals that the permeation height of the P-100 group with a smaller particle size is lower than that of the P-50 series with a larger particle size; this result indicates that the particle size of SAP has some influence on the permeability of concrete. The addition of SAP with a small dosage and a small particle size can improve the impermeability of concrete more obviously.

### 3.3. Influence of Porosity on Permeability

**Figure 7:**
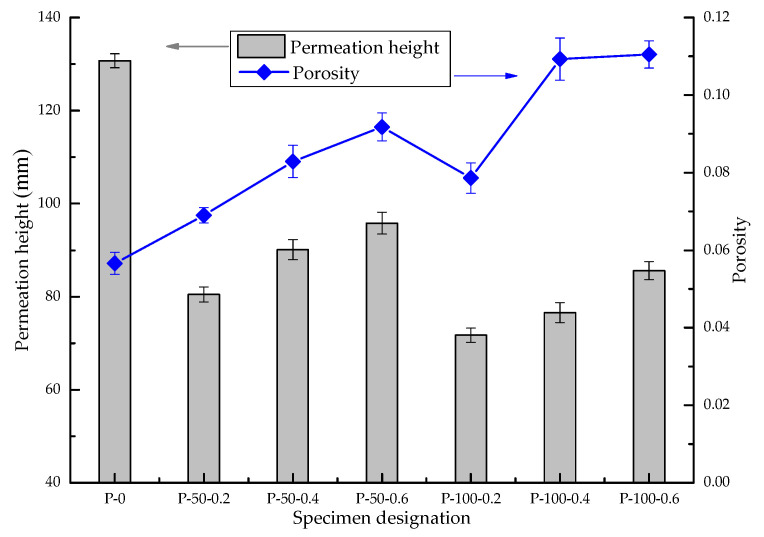
Relationship between permeation height and porosity.

Based on the results of the MIP experiment, the porosity of SAP concrete was statistically analyzed to study the effect of porosity on the permeability of SAP concrete, as shown in Figure 7. From Figure 7, the porosity of each sample is observed to increase as the amount of added SAP increases, and the porosity decreases as the SAP particle size decreases. There is no simple functional relationship between the permeability of concrete and the total porosity. The permeability of each specimen was tested by the hydraulic pressure method [24,25], and the microscopic pore structure was measured using the MIP experiment; the test results are shown in Figure 7.

Figure 7 shows that the porosity of the concrete increases with the increase in SAP content and it remains higher than that of the standard sample without SAP addition. The permeation height of SAP increases with the increase in SAP content but remains lower than that of the standard sample without SAP addition. It can be seen from the above phenomenon that the internal porosity of concrete has been greatly improved after SAP is added to the concrete. However, due to the poor connectivity of the internal pores, the penetration height of the concrete has not increased compared with the standard sample.

After adding SAP, the porosity of the concrete increased, whereas the penetration height decreased—that is, the impermeability increased. In the case of the addition of the same SAP concentration, the improvements in the porosity and anti-permeability of the small-diameter SAP concrete sample is more is more obvious.

### 3.4. Average Pore Size

The average pore size, which is an important parameter of the concrete pore structure, characterizes the pore structure of the overall situation. Han Yong Moon [26] studied the relationship between average pore diameter and chloride diffusivity in various concretes; their results show that there is a strong correlation between the chloride diffusivity of concrete and the average pore diameter, and the correlation coefficient can exceed 0.91. The chloride permeability coefficient increases with the increase in the average pore size, i.e., the average pore size can be used as the main parameter of concrete permeability. In general, the common concrete penetration resistance decreases with the increase in the average pore size.

**Figure 8:**
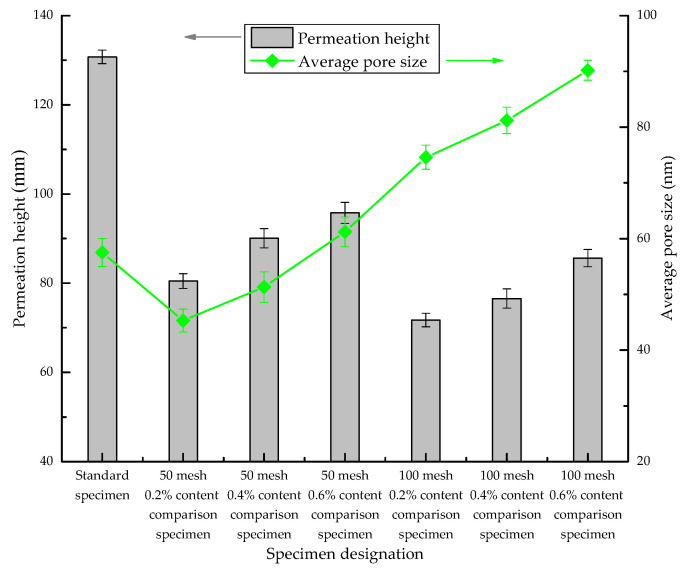
Comparison of the average pore size.

The average pore size comparison figure is drawn from the MIP experimental data, as shown in Figure 8. It can be seen from Figure 8 that the average pore sizes of the P-50 and P-100 samples increase with the increase in the SAP concentration and decrease with the decrease in the SAP particle size.

From the average pore size data, the permeability of SAP concrete does not decrease with the increase in the average pore size; rather, it shows a growing trend, which represents the unique performance of SAP in terms of its effect on the permeability of concrete.

## 4. Pore Structure Characteristics of SAP Concrete

Based on the water penetration under pressure test, SAP is found to have some influence on the permeability of concrete; however, its influence mechanism cannot be obtained and explained directly from the water penetration under pressure test. It is necessary to further analyze the pore structure of SAP concrete by means of scanning electron microscopy (SEM); the mechanism underlying the effect of SAP on the permeability of concrete will be discussed based on the microscopic pore structure characteristics.

The characteristics of the pore structure of concrete determine the permeability of concrete, and the change in pores has a significant influence on the permeability of concrete, from both macroscopic and microscopic perspectives. There is a certain relationship between the permeability and the connectivity of the internal pores. Only when the pore size is greater than the critical diameter will infiltration further proceed [27]. 

With the continuous improvement of pore measurement technology, the combination of computers and electron microscopes resulted in the development of a quantitative stereo micrography technique based on the optical microscopy image analysis method. Using this method, a series of parameters, including pore diameter, stomatal gradation, and stomatal distribution, can be obtained. 

In this paper, a large number of SEM photographs of concrete was obtained using scanning electron microscopy (SEM); the stomatal structure parameters of the samples were obtained by analyzing the micrographs with Image-Pro software. A TDCL-SU1510 scanning electron microscope was used to obtain the SEM micrographs of the microstructures. Finally, the micro-images were analyzed and processed with Image-Pro software to obtain the required pore parameters.

### 4.1. On the Influence of Pore Pressure Values

According to the Cantor equation [28], the capillary pressure *ρ* (units: Pa), surface tension *γ* (units: N/m), wet angle *θ* (units: °), and pore radius *r* (units: m) have the following relationship:

(3) $$\rho =\frac{2\gamma \cos\theta }{r}$$

**Figure 9:**
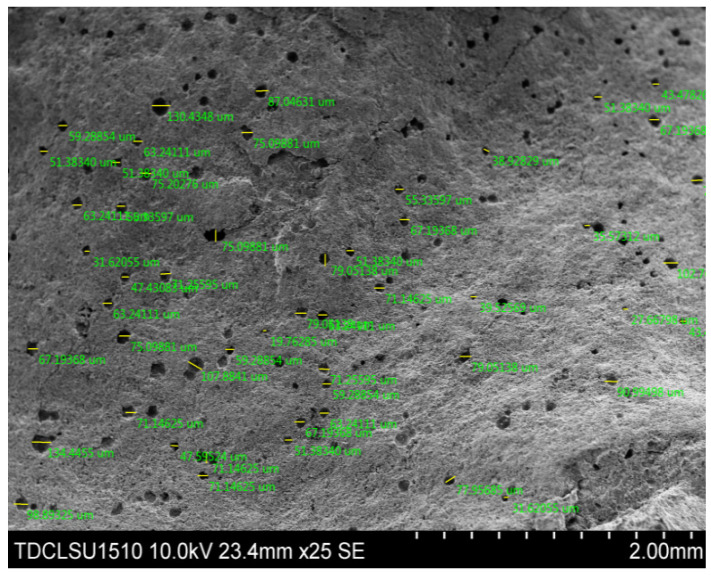
SAP bubble group in concrete.

SAP particles can also be considered as a group of air bubbles in concrete, which can not only block the connectivity of the pores but also increase the average concrete pore size, as shown in Figure 9. From Formula (3), the capillary pressure is inversely proportional to the pore radius, i.e., the smaller the pore size is, the greater the capillary pressure, the deeper the liquid inhalation, and the worse the impermeability. In many cases, the effect of capillary pressure on the impermeability of concrete is much greater than that of water pressure on impermeability. 

In fact, as long as the concrete is in contact with water, even if there is no water pressure difference, the phenomenon of infiltration will occur because of the role of capillary pressure. The increase in the average pore size causes the capillary pressure to decrease, which results in a shallow suction of the liquid and an increase in the impermeability. A decrease in capillary pressure (*ρ*) can reduce the permeability of concrete. Thus, we can preliminarily explain the phenomenon previously reported in Section 3.4—that is, the increase in the average pore size does not cause the increase in the penetration height.

### 4.2. On the Influence of Capillary Resistance

When the concrete is submerged in water, it will be affected by the combined effect of water pressure difference, capillary pressure, and capillary resistance. The following formula can fully reflect the relationship between the penetration rate and the water pressure, capillary pressure, and capillary resistance [29].

(4) $$\frac{dq}{dt}=\frac{K^{\prime }A\left(\Delta h+\rho -h_{f}\right)}{L^{\prime }}$$

where d*q*/d*t* represents the penetration rate (units: cm^3^/h), *K*’ represents the real permeability (units: cm/h), *A* represents the cross-section area of the specimen (units: cm^2^), Δh represents the water pressure difference acting on the surface of the specimen (units: cm)*, ρ* represents the capillary pressure (units: cm), *h_f_* represents the capillary resistance (units: cm), and *L*’ represents the actual penetration depth (units: cm).

With the increase of the actual penetration depth, the capillary resistance will change. When the penetration depth reaches the opposite side of the concrete specimen, the direction of the capillary pressure will also change—that is, from positive to negative (opposite to the water pressure). The capillary pressure, which originally promotes the penetration of the liquid, will hinder the penetration of the liquid in this case. Therefore, the real permeability coefficient calculated using Equation (4) varies nonlinearly with the actual penetration depth. The permeability and capillary permeability of concrete also have such a close relationship through the change in the relatively high capillary resistance. When the capillary penetration is poor, the capillary resistance is relatively high. 

**Figure 10:**
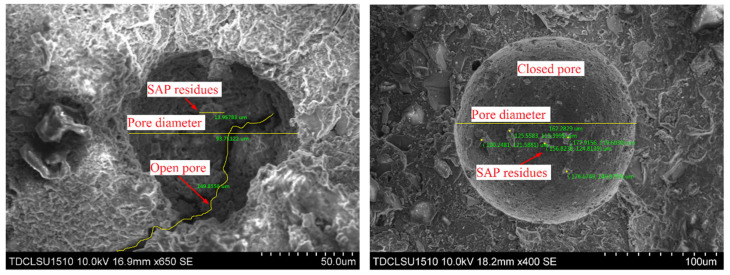
Single cavity connection (SEM).

Microscopic pore structure observation of each sample is conducted using SEM, and a typical internal cavity communicating state is obtained (as shown in Figure 10). Figure 10 shows that a large number of closed or semi-closed pores are formed because of the presence of SAP particles in the concrete, thereby inhibiting communication between the pores.

The tortuosity of the channel is an important parameter describing the seepage channel, which is defined as the ratio of the actual length of the seepage channel to the apparent length (macro distance) of the seepage medium—that is, the true length of the trajectory of the seepage fluid particles passing through the medium unit distance in the channel. The tortuosity of the channel is a dimensionless unit. The larger the value, the greater the number of mesopores and small pores or the better the connectivity of the pores, which can be calculated by mercury withdrawal in the MIP experiment [30,31,32].

**Figure 11:**
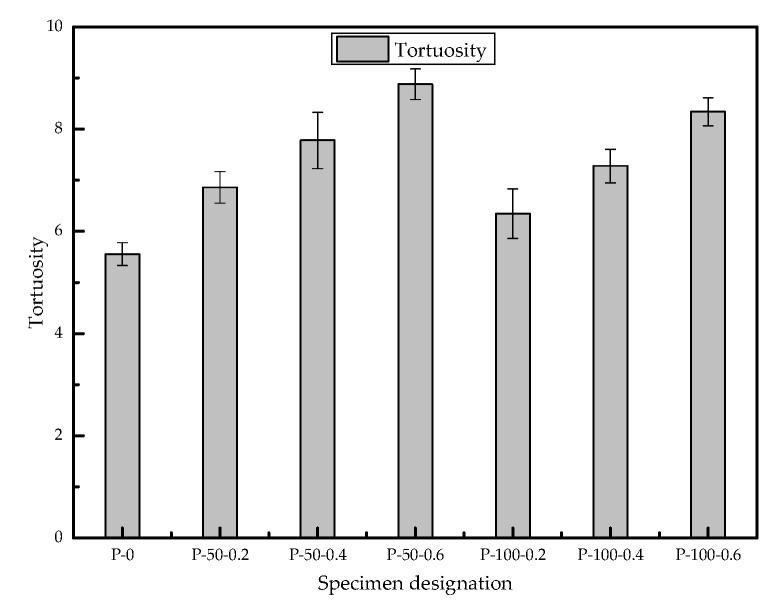
Comparison of the tortuosity of each sample group.

The analysis results of the porosity of the sample are shown in Figure 11. The tortuosity values of each sample increased after adding SAP, and all are higher than that of the standard sample without the addition of SAP. For the same particle size, the tortuosity value increases with the increase in SAP; this indicates that an increase in the amount of SAP will increase the value of the tortuosity of the concrete pores. In the case of the same SAP addition amount, the tortuosity value of the large-diameter SAP specimen is higher than that of the small-diameter SAP specimen.

**Figure 12:**
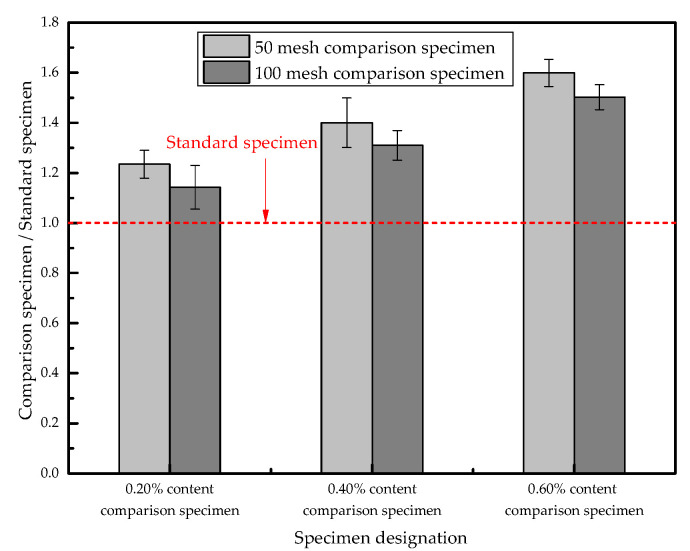
Normalized graph of sample tortuosity.

The normalized values of the tortuosity of the P-50 and P-100 samples are obtained by using the P-0 samples as the reference values; the normalized diagram of the tortuosity value of each sample is shown in Figure 12. In the case of the same amount of SAP added, the tortuosity value of the SAP small particle size sample is slightly higher, demonstrating that SAP with a smaller particle size is more likely to change the pore zigzag distribution inside concrete and increase its tortuosity value. 

From the above analysis, it can be seen that SAP particles effectively improve the capillary resistance—that is, the value of *h_f_* in Equation (4)—thereby effectively reducing the permeability of concrete.

### 4.3. “Threshold Effect” in SAP Particle Penetration

Rose [14] categorized the movement of water in porous material into seven stages. The water enters into the pores of the material by adsorption and surface diffusion. Liquid flow occurs according to Darcy’s law. The permeability of concrete materials is the process of capillary water seepage saturation and pressure penetration. Water infiltration is first caused by capillary action and then increases with the increase in internal water pressure. The driving force of water infiltration originates from the evaporation of water, capillary action, and pressure difference; under the action of the above-mentioned factors, the water continuously transmits and penetrates into the concrete. With the deepening of water transport, the friction between water and the capillary wall increases, and the water penetration rate decreases proportionally as the penetration depth increases.

**Figure 13:**
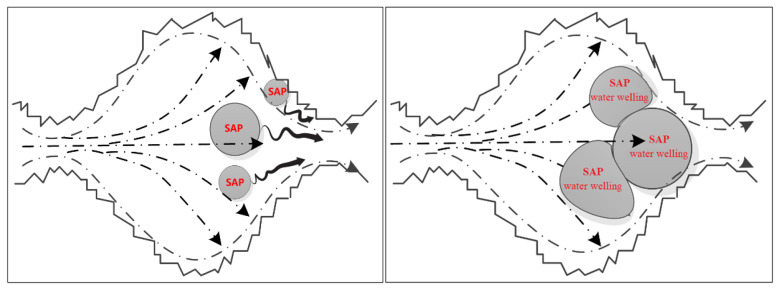
The schematic diagram of the “threshold effect”.

SAP concrete is different from ordinary porous concrete, which is formed by filling gas and foam; after the completion of hydration, each cavity will still contain SAP particle residues. Since the SAP particles have the characteristic of repeated water absorption, after permeating water re-enters, the SAP particles gradually absorb water and swell and then move toward the neck of the cavity under the action of water pressure until they finally block the cavity. This process will increase the resistance of liquid seepage to a certain extent and further promote the increase in capillary resistance, forming a “threshold effect” inside the concrete and reducing the capillary liquid surface tension; as a result, the impermeability of concrete is improved, as shown in Figure 13.

From the above analysis, it is known that the addition of SAP reduces the capillary pressure and increases the resistance of the pores. In addition, SAP also has a unique “threshold effect” in the cavity, which leads to the permeability of the SAP concrete still being good under the condition of increasing porosity and average pore diameter.

## 5. Conclusions

(1) After adding SAP of 0.2%~0.6% cement in concrete, its penetration height value will be reduced by 35%~45%. The penetration height of concrete specimens with the same SAP particle size will increase with the increase in SAP addition, and the penetration height of concrete specimens with the same SAP amount will decrease with the decrease in SAP particle size. This shows that, after adding SAP to concrete, its impermeability performance is improved to a certain extent, and the smaller SAP particle size and smaller addition amount have the best effect on improving the impermeability of concrete.

(2) Quantities of 0.2%~0.6% of SAP can increase the porosity of concrete from 0.056 to 0.069~0.111. The porosity and average pore diameter of concrete both increase greatly with the increase in SAP addition, and under the same SAP addition amount, the porosity and average pore diameter of SAP concrete with small particle diameters are higher.

(3) The addition of 0.2%~0.6% of SAP can increase the tortuosity of concrete by 1.14~1.5 times, and the tortuosity of concrete increases with the increase in SAP. Among them, the larger SAP particle size concrete specimens increased slightly.

(4) The addition of SAP decreases the internal capillary pressure of concrete; moreover, a large number of closed or semi-closed cavities, which are formed by the SAP particles in the concrete, cut off the connectivity of the pores, thereby increasing the impermeability of concrete. This explains the inherent mechanism of improvement of the impermeability of concrete in the case of SAP concrete with increasing porosity and average pore size. SAP has a unique “threshold effect” in the cavity, indicating that the permeability of the SAP concrete is still good under the condition of increasing porosity and average pore diameter.

## Figures and Tables

**Table 1:** Performance index of SAP for testing.

Performance Index	Unit	H-50	H-100
appearance	particle size	50 mesh	100 mesh
bulk density	g/mL	0.65~0.85	0.65~0.85
water absorption rate	multiple	400	700
water content	%	≤8	≤8
deionized water absorption	g/g	≥300	≥700
deionized water absorption rate (1:100)	s	≤50	≤65
0.9% NaCl water absorption	g/g	≥40	≥60
0.9% NaCl water retention capacity	g/g	≥45	≥40
0.9% NaCl absorption pressure (60 min, 0.3 psi)	g/g	≥25	≥27
pH	---	6.5~8.0	6.5~8.0

**Table 2:** Mix proportion of concrete.

Name	Cement (425 Silicate)/kg	Sand (0.25 mm)/kg	Pebble/kg	Water/kg	Water–Cement Ratio (*W*/*C*)	Additional Water Diversion (*W_e_*)/kg
Mix proportion	550	700	1045	165	$P_{W/C}=0.3$	29.7

**Table 3:** Grouping and numbering of the specimens.

Designation	Specimen Number	Quantity	W/C	SAP Particle Size (mesh)	Amount of SAP	Porosity
Standard specimen	P-0	6	0.30	0	0	5.66%
50 mesh 0.2% content comparison specimen	P-50-0.2%	6	0.30	50	0.2%	6.90%
50 mesh 0.4% content comparison specimen	P-50-0.4%	6	0.30	50	0.4%	8.29%
50 mesh 0.6% content comparison specimen	P-50-0.6%	6	0.30	50	0.6%	9.18%
100 mesh 0.2% content comparison specimen	P-100-0.2%	6	0.30	100	0.2%	7.86%
100 mesh 0.4% content comparison specimen	P-100-0.4%	6	0.30	100	0.4%	10.93%
100 mesh 0.6% content comparison specimen	P-100-0.6%	6	0.30	100	0.6%	11.05%

Note: For the numbers in the form of P-Y-Z, P denotes the mix proportion of concrete, Y is the particle size of the SAP, and Z is the amount of SAP.
